# Children’s rearfoot and midfoot motion while walking in school shoes

**DOI:** 10.1186/1757-1146-4-S1-O49

**Published:** 2011-05-20

**Authors:** Caleb Wegener, Richard Smith, Adrienne Hunt, Benedicte Vanwanseele, Andrew Greene, Joshua Burns

**Affiliations:** 1 Discipline of Exercise and Sports Science, Faculty of Health Sciences, The University of Sydney, NSW, 1825, Australia; 2 Faculty of Health Sciences, The University of Sydney/ Institute for Neuroscience and Muscle Research, The Children’s Hospital at Westmead, Sydney, NSW, 2145, Australia

## Background

Parents, health professionals and shoe manufacturers expect that shoes will not impede a child’s normal foot function or motor development. The aim of the current study was to determine the effect of school shoes on the rearfoot and midfoot motion of healthy children while walking.

## Methods

Twelve children performed five walking trials at a self selected velocity while barefoot and wearing school shoes (Daytona, Clarks). A 10 camera 200Hz 3D motion analysis system (EVaRT5.0, MAC) was used to calculate marker trajectories. Force plate data were collected at 1000Hz (Kistler™). Retro-reflective markers were attached to the right leg and to the foot/shoe at the navicular, 1^st^ and 5^th^ metatarsal heads and hallux. Rearfoot motion was measured with a wand marker cluster through a window in the shoe. A standing reference trial was used to embed segment axes and thence to calculate motion of the distal segment relative to the proximal segment. Data were normalised to the stance phase which was sub-divided from the anterior/posterior force data as: contact period (initial contact - maximum negative force); midstance (maximum negative force - zero) and propulsion (positive force - toe-off).

## Results

Five boys and seven girls participated in the study (mean age 9 years, range 5-13 years). During the contact period shoes decreased midfoot range of motion (ROM) in the frontal plane from 3.4° to 1.7° (p=0.002) and in the transverse plane from 22.0° to 11.6° (p<0.001). No significant difference in ROM occurred during midstance at either the rearfoot or midfoot. During propulsion shoes reduced rearfoot ROM in the frontal plane from 12.0° to 9.6° (p=0.026) and midfoot ROM in the sagittal plan from 19.6° to 10.8° (p<0.001) and in the transverse plan from 10.1° to 4.3° (p<0.001).

## Conclusions

Traditional school shoes restrict children’s foot motion during walking particularly at the midfoot during the contact period and propulsion phases of gait. The medium and long-term impacts of these changes are the focus of further research. The impact of school shoes on foot motion should be considered when assessing the paediatric patient and evaluating the effect of shoe or in-shoe interventions.

